# Low Rates of Co-Administration of Respiratory Vaccines Across the United States: Experiences from a Large Vaccination Ecosystem

**DOI:** 10.3390/vaccines14100844

**Published:** 2026-09-24

**Authors:** Ruth Carrico, Chris Kemp, Salwa Rashid

**Affiliations:** 1 Division of Infectious Diseases, School of Medicine, University of Louisville, Louisville, KY 40202, USA; 2 Data Analytics, VaxCare, LLC, Orlando, FL 32801, USA; ckemp@vaxcare.com; 3 Epidemiology and Population Health, School of Public Health and Information Sciences, University of Louisville, Louisville, KY 40202, USA; rashid08@gmail.com

**Keywords:** adult vaccination, pediatric vaccination, vaccine co-administration, respiratory vaccines, missed opportunities

## Abstract

Introduction: Giving more than one vaccine at a single visit is safe and is recommended by the Advisory Committee on Immunization Practices, but how often it happens in routine practice is not well described. We measured co-administration of influenza, COVID-19, and respiratory syncytial virus (RSV) vaccines where vaccine supply and on-site availability were consistent. Objective: To better characterize rates of co-administration and potential missed opportunities. Methods: We conducted a retrospective, cross-sectional analysis of records from a vaccine management platform serving more than 5000 clinics in 43 US states. Patients were age-eligible for at least one of the three vaccines, had a visit between 1 July 2023 and 30 April 2024, and received at least one. Those receiving two or more vaccines at one visit were compared with those receiving one using chi-squared tests. Multivariable logistic regression identified independent predictors of co-administration. Results: Of the 1,233,349 patients who received a respiratory vaccine, 50,582 active patients (4.1%) received two or more at the same visit. Rates were highest at health departments (13.4%), among Vaccines for Children (5.9%) and Medicaid (5.5%) beneficiaries, at the highest-volume clinics (5.2%), and among patients aged 51 to 64 years (5.0%). Influenza with COVID-19 accounted for 91.3% of co-administrations, influenza with RSV for 5.4%, and RSV with COVID-19 for 2.5%; 0.7% received all three. Cohorts differed by age, gender, insurance, clinic specialty, and clinic volume (all *p* < 0.0001). After multivariable adjustment, health department specialty remained the strongest independent predictor of co-administration with smaller independent associations for age, gender, insurance type, and clinic volume. Conclusions: Co-administration remained uncommon even where all three vaccines were stocked and available at the visit, so supply does not explain this shortfall. The settings and payer groups at either end of the range show where standing orders and visit prompts could recover missed opportunities.

## 1. Introduction

Simultaneous vaccine administration, or co-administration, is the delivery of more than one vaccine using different syringes and anatomic sites at the same clinic visit [1]. Co-administration is considered an important strategy for increasing vaccination coverage across populations and has become essential in reducing the number of healthcare visits, improving compliance with immunization schedules, and enhancing timely protection against diseases that may be preventable through vaccination [2,3]. With only rare exceptions, co-administered vaccines have similar immunogenicity and adverse event rates compared with vaccines administered alone [4,5,6]. Key experimental evidence supports co-administration in both pediatric and adult populations. For example, in a comprehensive review of 34 studies (approximately 21,000 participants) evaluating the hexavalent DTaP-HBV-Hib vaccine co-administered with 12 different infant vaccines (pneumococcal conjugate, meningococcal, rotavirus, MMRV), the study found high seroprotection rates with no clinically significant immune interference [7]. A systematic review of 26 pediatric studies found a modestly increased risk of any adverse event (OR: 1.54) but no significant increase in serious adverse events (OR: 1.07, 95% CI: 0.92–1.25) [8]. A multicentered RCT of 593 18 month old Chinese children demonstrated non-inferior immunogenicity when IPV, DTaP, and hepatitis A vaccines were co-administered when compared with separate administration. There were comparable antibody titers across all antigens, and no serious adverse events were identified [9]. Among adults, a UK-based multicentered randomized phase 4 study of 679 adults [the ComFluCOV trial] evaluated six combinations of COVID-19 vaccines with influenza vaccines. Co-administration raised no safety concerns, resulted in acceptable reactogenicity, and preserved binding antibody responses for both vaccines [10]. A phase 4 RCT of 1152 Chinese adults demonstrated non-inferior immunogenicity when a COVID-19 vaccine was co-administered with IIV4 and PPV23. A 100% seroconversion rate for SARS-CoV-2 neutralizing antibodies was reported in the co-administration group [11]. Recommendations from the Centers for Disease Control and Prevention (CDC) Advisory Committee on Immunization Practices (ACIP) support co-administration of most indicated vaccines and explicitly endorse co-administration of vaccines intended to protect against respiratory viruses (e.g., influenza, respiratory syncytial virus [RSV], and COVID-19) in people of all ages [12,13].

Despite the extensive data supporting the safety and efficacy of co-administered vaccines and the recommendations from the CDC, missed opportunities for co-administration are prevalent in routine clinical practice [14,15,16]. Studies show that caregiver, patient, and provider concerns about co-administration are common [17,18]. However, more data are needed to identify motivators of co-administration acceptance and hesitance in order to guide interventions that may increase vaccine uptake across the lifespan. These interventions are particularly crucial for vulnerable populations who have lower vaccine coverage rates despite being at higher risk for complications from vaccine-preventable illnesses [19,20,21,22,23]. The importance of understanding vaccine uptake in this context is underscored by the 2023–2024 respiratory virus season marking a critical juncture in vaccine delivery, representing the first season when vaccines against influenza, COVID-19, and RSV were simultaneously available for adults aged 60 years and older. This unique opportunity for comprehensive respiratory virus protection through co-administration occurred against the backdrop of ongoing concerns about concurrent circulation of multiple respiratory pathogens (i.e., the ‘tripledemic’).

To better characterize rates of co-administration and potential missed opportunities, this study leveraged data from a large vaccine ecosystem that provides end-to-end vaccine management for more than 5000 clinics located in 43 states across the United States. The large and diverse geographic presence and variety of clinics using VaxCare provide an unusual opportunity to evaluate co-administration when the hurdle of availability and access to vaccines and vaccination has been removed.

The objectives of this study were to (1) describe the frequency of co-administration of COVID-19, influenza, or RSV vaccines within the large vaccine ecosystem during the 2022–2023 respiratory virus season and (2) determine whether sociodemographic factors and clinic attributes contribute to co-administration of these vaccines within that same vaccine ecosystem.

## 2. Methods

### 2.1. Study Design

This retrospective, observational descriptive study analyzed data from a large, end-to-end vaccine ecosystem (VaxCare LLC, Orlando, FL, USA). This vaccination network has been previously described and includes more than 5000 active clinic sites across more than 40 US states. In 2023, the more than 17,000 providers in this network administered approximately 4 million vaccines [24]. Through the ecosystem’s automated technology platform, which is integrated with clinic electronic health record (EHR) systems, vaccine encounters are documented, billed to insurance, and reimbursed, resulting in a robust repository of vaccination information. Providers and clinics within this ecosystem can select the vaccines they would like to offer to their patient populations. Vaccine inventory is continuously monitored and replenished, making vaccines available for immediate access at the time of the patient visit.

This study included data from active patients, defined as those who were eligible for vaccination with at least one of three respiratory vaccines (COVID-19, influenza, and RSV), had a clinic visit between 1 July 2023 and 30 April 2024, and received one or more of those vaccines during the study period. Active patients were categorized according to the number and types of those vaccines received.

The study was approved by the University of Louisville Institutional Review Board [#23.0719]. Data were provided by the VaxCare data science team and all identifiers were removed. The IRB waived the requirement for informed consent due to the retrospective review of de-identified data.

### 2.2. Statistical Analysis

Demographic and clinic data for the overall population and age-group subpopulations are presented as percentages of their respective totals. Statistical analyses were performed to compare characteristics among active patients who received 1 respiratory vaccine with those who received at least 2 respiratory vaccines at a single visit (co-administration). Additionally, characteristics for patients who received co-administered vaccines were compared for those who received 2 vaccines and those who received at least 3 vaccines at a single visit. We also built a multivariable logistic regression model to assess the independent contribution of age group, gender, insurance type, specialty type, and annual clinic vaccination volume on co-administration status. Frequencies and percentages were used to summarize categorical variables. Characteristics were statistically compared between the two groups using Chi-squared tests. *p* values of <0.05 were considered statistically significant. Analyses were performed using SAS 9.4 software (SAS Institute Inc., Cary, NC, USA).

## 3. Results

### 3.1. Study Population

The vaccine ecosystem had a total of 1,850,269 patients who were vaccinated with any vaccine, and of them, 1,233,349 (67%) received one or more respiratory vaccines during the study period (Figure 1). Of the study population, 40.5% were 65 years or older, and 56.4% were female. Most patients were covered by commercial insurance (53%) or Medicare (39.9%), with a smaller proportion covered by Medicaid or the Vaccines for Children (VFC) program. The most common clinic sites for care were internal medicine (33.9%), pediatric (33.1%), and family medicine (25.1%).

Within the vaccination ecosystem, clinics are categorized based on their annual vaccination volume: highest (>10,000 vaccines annually), extra high (between 2001 and 10,000 vaccines annually), high (1000 to 2000 vaccines annually), medium (401 to 999 vaccines annually), and low (≤400 vaccines annually). Clinics in the highest volume bracket are typically health system clinics and provided vaccines to nearly one-quarter of the study population.

### 3.2. Co-Administration of Respiratory Vaccines

Of those with a respiratory vaccine, most active patients in the study population (*n* = 1,182,767; 95.9%) received a single vaccine only (no co-administration). Only 50,582 active patients (4.1%) received 2 or more co-administered respiratory vaccines.

### 3.3. Differences in Characteristics Between Cohorts

Across all subgroups studied, co-administration rates were substantially lower than single-vaccination rates. The subgroup with the highest co-administration rate was the population of active patients vaccinated at health department sites (13.4%), followed by those with VFC coverage (5.9%), with Medicaid coverage (5.5%), receiving vaccines at the highest-volume clinics (5.2%), and aged 51 to 64 years (5.0%).

The differences in subgroup distribution between the co-administration and no co-administration cohorts are shown in Table 1. The distributions of patient ages, patient genders, insurance types, clinic types, and clinic vaccine volumes were significantly different between the cohorts receiving co-administration versus no co-administration (all *p* < 0.0001). The largest differences between the co-administration and no co-administration cohorts were by clinic specialty, with higher proportions of patients vaccinated at health departments receiving co-administered vaccines (between-group difference, +18.1 percentage points) and lower proportions of patients vaccinated in pediatric clinics receiving co-administered vaccines (−9.0 percentage points). When the distribution of active patients by clinic vaccine volume was compared between cohorts, the differences between the co-administration and no co-administration cohorts became increasingly disparate with decreasing vaccine volume, from +7.0 percentage points in the highest-volume clinics to −5.2 percentage points in low-volume clinics. Additionally, compared with the no co-administration cohort, the co-administration cohort had higher proportions of patients aged 51–64 years (between-group difference, +4.3 percentage points) and lower proportions of patients 65 years or older (−5.2 percentage points).

#### Multivariable Analysis of Co-Administration

Table 2 presents the multivariable logistic regression and analysis of co-administration, along with model goodness-of-fit and specification diagnostics. In this model, the health department remained by far the strongest independent predictor of co-administration (adjusted odds ratio [aOR]: 4.41, 95% CI: 3.89–5.01, *p* < 0.001), consistent with the univariate findings shown in Table 1. Age, gender, insurance type, and clinic vaccine volume each remained statistically significant independent predictors of co-administration after adjustment for specialty type (all *p* < 0.05, with the exception of age 51–64 years [aOR: 1.08, 955 CI: 0.96–1.22, *p* = 0.222]), indicating that these associations were not simply attributable to the uneven distribution of patient characteristics across specialty types.

Model fit and specification of the multivariable logistic regression were assessed using standard diagnostic tests. The Hosmer–Lemeshow goodness-of-fit test indicated no evidence of lack of fit (χ^2^ = 10.34, df = 8, *p* = 0.24) supporting adequate calibration of predicted probabilities across risk deciles. The model demonstrated modest discrimination, with an area under the receiver operating characteristic curve (AUC/c-statistic) of 0.66. Pregibon’s link test for model specification was non-significant for the squared linear predictor term (_hatsq: *p* = 0.18), providing no evidence of omitted variables or incorrect link-function specification. The overall model was statistically significant compared with the null model (likelihood ratio χ^2^(16) = 844.2, *p* < 0.001; McFadden’s pseudo-R^2^ = 0.05, consistent with the modest discrimination expected from demographic and clinic-level predictors alone, without clinical or behavioral covariates).

Beyond the pronounced effect of health department specialty, Table 2 demonstrates several additional independent associations with co-administration after multivariable adjustment. Compared with patients 19–50 years of age, children 6 months-2 years old had lower adjusted odds of co-administration (aOR: 0.62, 95% CI: 0.51–0.75), as did adults 65 years and older (aOR: 0.76, 95% CI: 0.67–0.87). Adults aged 51–64 years did not differ significantly from the reference group after adjustment (aOR: 1.07, 95% CI: 0.93–1.22), and children 3–18 years showed a similar but non-significant trend toward lower odds (aOR: 0.86, 95% CI: 0.72–1.01, *p* = 0.065). Males had modestly higher adjusted odds of co-administration than females (aOR: 1.22, 95% CI: 1.12–1.32). Relative to commercially insured patients, co-administration was more likely among Medicaid (aOR: 1.30, 95% CI: 1.04–1.61) and Vaccines for Children (VFC) program coverage (aOR: 1.41, 95% CI: 1.13–1.75), whereas Medicare insured patients had lower adjusted odds of co-administration (aOR: 0.78, 95% CI: 0.70–0.87). Within specialty type, family medicine did not differ significantly from internal medicine (aOR: 1.08, 95% CI: 0.94–1.24), whereas OB/GYN (aOR: 0.57, 95% CI: 0.39–0.85) and pediatric clinics (aOR: 0.88, 95% CI: 0.79–0.98) had significantly lower adjusted odds of co-administration. Finally, the adjusted odds of co-administration increased progressively with annual clinic volume, from an aOR of 0.74 (95% CI: 0.63–0.86) in low-volume clinics to an aOR of 1.74 (95% CI: 1.53–1.99) in the highest volume clinics, relative to medium volume clinics.

### 3.4. Co-Administration by Vaccine Type

In the co-administration cohort, the most commonly co-administered respiratory vaccines were influenza and COVID-19 (91.3%). In addition, 5.4% of co-administration events included influenza and RSV, and 2.5% of co-administration events included RSV and COVID-19 vaccines. Only 0.7% of patients in the co-administration cohort received all 3 respiratory vaccines simultaneously.

The co-administered vaccine combinations were evaluated by age group, gender, insurance type, specialty type, and clinic vaccination volume. In general, adults 51 years and older received more co-administered influenza and RSV vaccines than patients in other age groups. Influenza and COVID-19 was the only co-administered combination used in those aged 6 months to 2 years and predominated in people aged 3 to 50 years.

### 3.5. Co-Administration by Number of Co-Administered Vaccines

Significantly more patients received 2 co-administered vaccines (99.3%) than >2 co-administered vaccines (0.7%). Most individuals receiving 3 co-administered vaccines were aged 51–64 years (67.5%) or 65 years and older (31.2%) and were vaccinated in clinics administering at least 2001 vaccines annually (extra high or highest volume; 73.2%) (Table 3). The distribution of patients receiving two versus more than two co-administered vaccines differed significantly by age group, insurance type, specialty type, and clinic vaccination volume (all *p* < 0.001). There was no statistically significant difference in the gender distribution of those who received two versus more than two co-administered vaccines (*p* = 0.299).

## 4. Discussion

In this study of a large, nationwide vaccine ecosystem, we found low rates of co-administration of respiratory vaccines. Eligibility for co-administration was driven by the CDC ACIP recommendation for the particular vaccine. For example, influenza vaccines are recommended for those 6 months of age and older, so an infant younger than 6 months would not be eligible for receipt of that vaccine. Individuals 50 years of age and older may be eligible for influenza, RSV, and COVID-19 vaccines so they would be included in the denominator as eligible. The study did not aim to determine whether vaccine selection and administration were appropriate for an individual patient. The study did aim to see which patients received co-administration when it could have been appropriate. Despite the CDC recommendations for co-administration of respiratory vaccines, fewer than 5% of vaccine visits included multiple vaccines, potentially indicating persistent missed opportunities for optimizing vaccination delivery in real-world clinical settings. To our knowledge, this is the first large study to examine vaccine co-administration in health clinics across multiple states and a wide range of clinic types and sizes.

The underutilization of co-administration represents a critical gap in adult immunization delivery, particularly given evidence supporting the safety, immunogenicity, and operational benefits of administering multiple vaccines simultaneously. Studies of influenza, COVID-19, and RSV vaccines have consistently shown that co-administration does not compromise immune responses or increase serious adverse events, supporting broader implementation in routine practice [9,10,11]. Co-administering vaccines reduces the number of healthcare visits required and addresses barriers such as limited patient access and clinic capacity constraints [8].

Our findings indicate that respiratory vaccine co-administration is more common and feasible among certain demographic groups and care settings. Middle-aged adults (51–64 years), men, commercially insured patients, and those vaccinated in primary care clinics and health departments were more likely to receive co-administered vaccines. These patterns are unlikely to be due to chance and likely reflect systematic differences in vaccination practices or patient preferences across different population segments. For example, differences in co-administration rates may be related to employee immunization or preventive health initiatives in the workplace among commercially insured and middle-aged adults, differences in health care-seeking behaviors between genders, and stronger vaccination infrastructure in high-volume clinics [25,26].

These interpretations are further supported by the multivariable analysis which showed that the differences in co-administration by age, gender, insurance type, and clinic volume persisted after adjustment for clinical specialty type. Although health department status was independently associated with substantially higher odds of co-administration, the smaller demographic and payer-related differences were not simply artifacts of this specialty effect, suggesting that multiple, at least partially independent factors influence co-administration practice.

The age-related pattern observed in the multivariable model (Table 2) may reflect a combination of vaccine eligibility and clinical scheduling practices. Because COVID-19 vaccine and RSV monoclonal antibody indications are age-restricted, young children under 2 years of age are eligible for fewer respiratory vaccines at a given visit than older patients, which automatically limits the opportunity for co-administration in this age group. A similar, if less pronounced pattern is plausible across childhood more broadly. The lower adjusted odds of co-administration among adults 65 years and older may reflect clinician or patient hesitation about administering multiple vaccines at a single visit given age-related comorbidities.

The modestly higher adjusted odds of co-administration among males relative to females is a notable finding that, to our knowledge, has not been well characterized in the co-administration literature. One possible explanation is that males, who generally attend fewer preventive care visits than females, may be more likely to receive multiple vaccines when they do present for care, effectively bundling vaccination into a less frequent visit pattern. Alternatively, this finding could reflect underly differences in the clinical context of visits by gender. Further study using visit-level data may help clarify the mechanism underlying this association.

The higher adjusted odds of co-administration among Medicaid and VFC-insured patients, and the lower adjusted odds among Medicare beneficiaries (Table 2) warrant separate consideration from the crude, unadjusted comparisons described above. Medicaid and VFC-eligible patients are disproportionately seen in safety net settings such as health departments where standing orders and integrated immunization workflows are common and may be specifically designed to bundle vaccines and reduce the number of visits required for populations who face greater barriers to returning for care. This systems-level explanation is consistent with the strong, independent effect of health department specialty identified in the same model. The lower adjusted odds among Medicare beneficiaries may relate to the historically fragmented coverage of adult vaccines across Medicare Part B and Part D, which has created administrative and billing complexity that could discourage co-administration. However, this may be less likely among VaxCare clinics due to the abilities of those settings to administer vaccines covered through those Medicare programs. We note that this adjusted pattern for Medicaid and VC coverage runs counter to the unadjusted, systems-barrier framing discussed elsewhere in this section (e.g., that low reimbursement and administrative burden discourage vaccine stocking in these populations); reconciling these two perspectives, potentially by examining stocking practices directly as well as patient-provider discussions, may be important elements for future research.

Within specialty type, the absence of a significant difference between family medicine and internal medicine after adjustment suggests that specialty, apart from the pronounced health department effect, has only a limited independent influence on co-administration. In contrast, the lower adjusted odds observed in OB/GYN clinics may reflect the vaccine safety concerns and hesitance present in that population. The modestly lower adjusted odds in pediatric clinics were somewhat unexpected given the field’s general reliance on structured, multi-vaccine immunization schedules. This finding may reflect the more constrained age-based eligibility for COVID-19 and RSV monoclonal antibody immunization in children, or clinician practices that intentionally space vaccines to limit injection burden or facilitate monitoring for reactogenicity in younger patients.

Finally, the strong, graded association between annual clinic vaccination volume and co-administration reinforces the broader interpretation, discussed above for health departments, that infrastructure and systems-level factors are central drivers of co-administration practice. Higher volume clinics are more likely to have implemented workflow processes that build upon those provided by the VaxCare systems (e.g., automated vaccine reorders and stocking, electronic medical record integration, insurance coverage verification, and management of billing), whereas low volume clinics may lack personnel dedicated to the vaccination process, making co-administration comparatively less feasible.

The higher rates of co-administration observed in specific settings—such as high-volume clinics and health departments—underscore the critical role that infrastructure and system supports play in optimizing vaccine delivery. Studies have consistently shown that implementing standing orders, integrated vaccination records, clinician reminders, and care coordinator support can substantially increase vaccine uptake relative to routine care [27]. Clinics with standing orders streamline workflows by authorizing non-provider staff to assess vaccine eligibility and administer vaccines without direct clinician involvement, a practice endorsed by the CDC and ACIP [28]. Similarly, centralized immunization registries and care coordinators facilitate the timely identification of vaccination needs and enable delivery of multiple vaccines during a single encounter [29]. These systems-level interventions may reduce missed opportunities for co-administration and promote efficient use of healthcare resources. In an analysis of Medicare claims data examining the extent of COVID-19 and influenza vaccine administration among older adults, Harris and colleagues observed that racial and ethnic differences in coadministration rates may be more related to access and awareness than to vaccine acceptance itself. Their findings further support attention to structural barriers and operational factors in order to influence vaccine acceptance and resultant co-administration [16].

Conversely, lower co-administration rates among pediatric clinics and low-volume sites suggest potential systemic barriers to vaccine co-administration. By contrast, Medicaid and VFC-insured patients showed significantly higher, not lower, adjusted odds of co-administration relative to commercially insured patients (Table 3). This pattern and its likely explanation are discussed further below. The disparities in co-administration by clinical setting and insurance type highlight areas where targeted interventions—such as improved provider training and streamlined clinic workflows—may help close the co-administration gap. Finally, differences in provider training and knowledge regarding contraindications could also explain the observed patterns. Vaccine co-administration may be enhanced through provider training, with a focus on vaccine safety, co-administration reassurance, and strategies for patient and caregiver counseling on co-administration to reduce vaccine hesitancy [30].

Further research into the causes of low co-administration rates and disparities in co-administration is needed. Future research should explore patient and provider perceptions of co-administration through qualitative studies. Additionally, the feasibility and acceptability of workflow redesign to incorporate co-administration protocols should be evaluated. Cost-effectiveness analyses examining the impact of improved co-administration on healthcare utilization and disease prevention are also warranted.

This study has limitations that are inherent to its retrospective design, which limits inferential analyses. Additionally, reliance on patient EHR data may incompletely capture patient demographics or vaccinations received outside the ecosystem (e.g., at pharmacies, workplaces, or community events). Lack of race and ethnicity data prevents analysis of disparities across these different groups, but the large sample size helps inform overall co-administration trends, which are likely robust across various ethnic and racial groups. Further, VaxCare is not yet available in all US states. Within states where VaxCare has a presence, rural clinic sites may be underrepresented. Finally, the dataset did not include information about contraindications, prior vaccinations, or provider-level decision making, so true eligibility for co-administration was not calculated. VaxCare clinics provide vaccines on-site, thereby facilitating access. Although patients can access vaccines through other routes (e.g., other clinics, pharmacies), the ability to seamlessly access co-administered vaccines increases co-administration opportunities and likely influences co-administration rates. The purpose of this study, however, was to report what has occurred in these clinics and serve as a baseline for improvement opportunities. The results also provide the chance to see co-administration rates under what could be considered a best-case access scenario. The exceptionally large sample size of over 1.2 million individuals allowed us to capture real-world vaccination practices across various demographics and settings, enhancing the external validity of the findings. Strengths of this study include the large sample size, geographic diversity, and real world settings, which capture actual clinical practice patterns.

An additional limitation is that the multivariable model did not account for clustering of patients within clinic sites, which may lead to underestimated standard errors. We also did not explicitly account for the recommended timing of influenza vaccination relative to other respiratory vaccines. ACIP recommends that influenza vaccination ideally be completed by the end of October to ensure protection before peak influenza circulation, whereas updated COVID-19 vaccine formulations lack a comparably narrow recommended window. This staggered guidance and rollout timing could reduce opportunities for co-administration for some patients independent of clinic or patient-level barriers. However, because our study period (1 July 2023–30 April 2024) spanned the full window in which all three respiratory vaccines were recommended and available, including the early fall rollout period, the observed low co-administration rate is unlikely to be explained primarily by a mismatch between the study period and vaccine recommendation timing. Timing misalignment at the individual patient level (e.g., early influenza vaccination in September before COVID-19 vaccine availability or patient eligibility) may nonetheless have contributed to missed co-administration opportunities.

## 5. Conclusions

This study highlights the possibility of missed opportunities for co-administration of respiratory vaccines in the United States and identifies population- and clinic-level factors associated with higher uptake. Notable disparities across age groups, gender, insurance types, specialty types, and vaccination volume demonstrate that both patient- and systems-level influences, such as access to care, provider practices, and clinic guidelines, shape co-administration patterns. Enhancing co-administration practices could improve vaccine coverage, reduce preventable illnesses, and streamline healthcare delivery.

Future efforts should focus on targeted interventions, including policy changes to address insurance-related disparities, expanding co-administration coverage across payer types, and implementing provider training programs to build confidence in co-administration practices across specialties. Additional research is needed to explore disparities by socioeconomic status, geographic regions, and race and ethnicity. Studies investigating short-term sequelae (e.g., adverse events) and long-term metrics (e.g., vaccine effectiveness, seroconversion, and the need for booster doses) could support efforts to increase co-administration rates by reassuring both patients and providers. Pilot studies on clinic workflow redesign, such as standing orders and integration of co-administration, coupled with cost-effectiveness analyses, could provide critical insights for improving vaccine coverage and optimizing resource use in diverse health care settings.

## Figures and Tables

**Figure 1:**
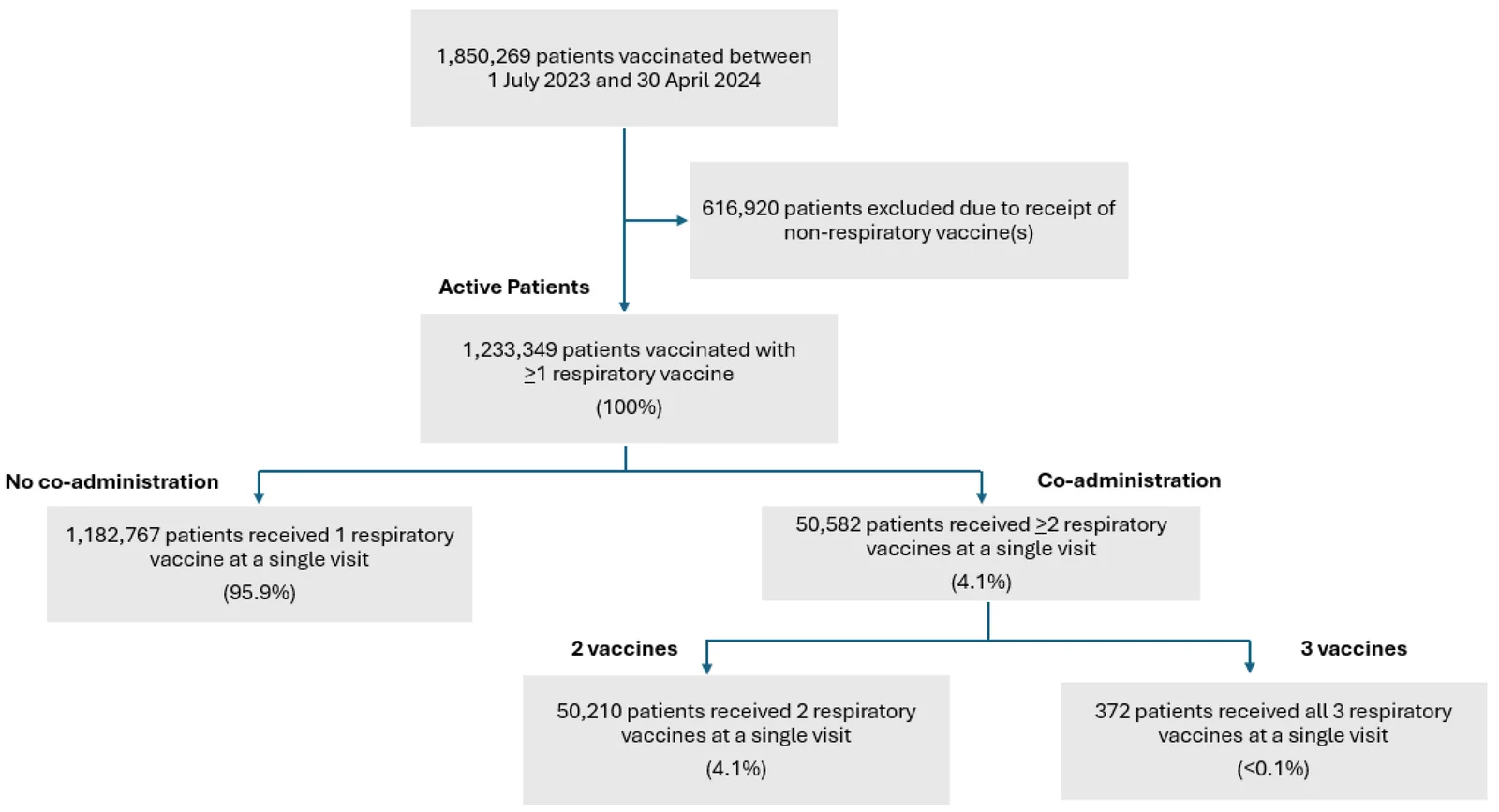
Flow diagram of study population identification and cohorting.

**Table 1:** Comparison Between Patients Who Received 1 Respiratory Vaccine (No Co-administration) or 2 or More Respiratory Vaccines (Co-administration) in a Nationwide Vaccine Ecosystem During the 2023–2024 Season.

Characteristic	All Active Patients *n* = 1,233,349		No Co-Administration (1 Respiratory Vaccine) *n* = 1,182,767		Co-Administration (≥2 Respiratory Vaccines) *n* = 50,582		Between-Group Difference (Co-Administration − No Co-Administration), Percentage Points	*p* Value
	*n*	%	*n*	%	*n*	%		
Age group								
6 months to 2 years	66,168	5.4	64,227	5.4	1941	3.8	−1.6	<0.0001
3−18 years	199,014	16.1	191,017	16.2	7997	15.8	−0.4	
19−50 years	240,087	19.5	228,835	19.4	11,252	22.3	2.9	
51−64 years	229,222	18.6	217,759	18.4	11,463	22.7	4.3	
≥65 years	498,858	40.5	480,929	40.7	17,929	35.5	−5.2	
Gender								
Woman	695,299	56.4	669,075	56.6	26,224	51.8	−4.8	<0.0001
Man	537,739	43.6	513,383	43.4	24,356	48.2	4.8	
Other	311	0.0	309	0.0	2	0.0	0	
Insurance type								
Commercial	657,394	53.3	628,537	53.1	28,857	57.1	4	<0.0001
Medicaid	44,552	3.6	42,109	3.6	2443	4.8	1.2	
Medicare	491,439	39.9	474,522	40.1	16,917	33.4	−6.7	
VFC	39,964	3.2	37,599	3.2	2365	4.7	1.5	
Specialty								
Family medicine	308,931	25.1	297,669	25.2	11,262	22.3	−2.9	<0.0001
Health department	93,515	7.6	80,939	6.8	12,576	24.9	18.1	
Internal medicine	417,876	33.9	403,570	34.1	14,306	28.3	−5.8	
OB/GYN	5110	0.4	5018	0.4	92	0.2	−0.2	
Pediatric	407,917	33.1	395,571	33.4	12,346	24.4	−9.0	
Annual vaccine volume								
Highest (>10,000)	294,823	23.9	279,349	23.6	15,474	30.6	7.0	<0.0001
Extra high (2001−10,000)	402,524	32.6	384,770	32.5	17,754	35.1	2.6	
High (1000−2000)	217,457	17.6	208,832	17.7	8625	17.1	−0.6	
Medium (401−999)	178,349	14.5	172,840	14.6	5509	10.9	−3.7	
Low (≤400)	140,196	11.4	136,976	11.6	3220	6.4	−5.2	

**Table 2:** Univariable and Multivariable Logistic Regression Analysis of Co-Administration.

Characteristic	Unadjusted OR	95% CI	*p*	Adjusted OR ^a^	95% CI	*p*
Age group						
19−50 years (reference)	1.00	Reference	—	1.00	Reference	—
6 months−2 years	0.62	0.48–0.79	<0.001	0.62	0.51–0.75	<0.0001
3−18 years	0.85	0.74–0.99	0.032	0.86	0.72–1.01	0.065
51−64 years	1.07	0.94–1.22	0.307	1.07	0.93–1.22	0.341
≥65 years	0.76	0.67–0.86	<0.001	0.76	0.67–0.87	<0.001
Gender						
Female (reference)	1.00	Reference	—	1.00	Reference	—
Male	1.21	1.11–1.32	<0.001	1.22	1.12–1.32	<0.001
Insurance type						
Commercial (reference)	1.00	Reference	—	1.00	Reference	—
Medicaid	1.27	1.03–1.57	0.028	1.30	1.04–1.61	0.021
Medicare	0.78	0.71–0.86	<0.001	0.78	0.70–0.87	<0.001
VFC	1.38	1.11–1.71	0.004	1.41	1.13–1.75	0.002
Specialty						
Internal Medicine (reference)	1.00	Reference	—	1.00	Reference	—
Family Medicine	1.07	0.94–1.21	0.298	1.08	0.94–1.24	0.304
Health department	4.38	3.87–4.97	<0.001	4.41	3.89–5.01	<0.001
OB/GYN	0.56	0.21–1.52	0.255	0.57	0.39–0.85	0.005
Pediatric	0.88	0.78–1.00	0.043	0.88	0.79–0.98	0.018
Annual vaccine volume						
Medium (401−999) (reference)	1.00	Reference	—	1.00	Reference	—
Highest (>10,000)	1.74	1.49–2.04	<0.001	1.74	1.53–1.99	<0.001
Extra high (2001−10,000)	1.45	1.24–1.69	<0.001	1.45	1.24–1.69	<0.001
High (1000−2000)	1.30	1.09–1.54	0.003	1.29	1.06–1.57	0.011
Low (≤400)	0.74	0.59–0.92	0.007	0.74	0.63–0.86	<0.001

^a^ Adjusted for age group, gender, insurance type, specialty, and annual clinic vaccination volume simultaneously; 95% CIs use cluster-robust standard errors clustered on specialty × volume category. Details on this proxy clustering approach is addressed in the Discussion. Reference categories: aged 19–50 years, female, commercial insurance, internal medicine, and medium clinic volume (401–999 vaccines annually). OR = odds ratio; CI = confidence interval.

**Table 3:** Comparison Between Patients Who Received 2 or More Than 2 Respiratory Vaccines in a Nationwide Vaccine Ecosystem During the 2023-2024 Season.

Characteristic	2 Respiratory Vaccines *n* = 50,210		>2 Respiratory Vaccines *n* = 372		Between-Group Difference (>2 Vaccines − 2 Vaccines), Percentage Points	*p* Value
	*n*	%	*n*	%		
Age group						
6 months to 2 years	1941	3.9	0	0.0	−3.9	<0.0001
3−18 years	7996	15.9	1	0.3	−15.6	
19−50 years	11,248	22.4	4	1.1	−21.3	
51−64 years	11,212	22.3	251	67.5	45.2	
≥65 years	17,813	35.5	116	31.2	−4.3	
Gender						
Female	26,046	51.9	178	47.9	−4.0	0.299
Male	24,162	48.1	194	52.2	4.1	
Other	2	0.0	0	0.0	0	
Insurance type						
Commercial	28,561	56.9	296	79.6	22.7	<0.0001
Medicaid	2400	4.8	43	11.6	6.8	
Medicare	16,885	33.6	32	8.6	−25.0	
VFC	2364	4.7	1	0.3	−4.4	
Specialty						
Family medicine	11,136	22.2	126	33.9	11.7	<0.0001
Health department	12,520	24.9	56	15.1	−9.8	
Internal medicine	14,189	28.3	117	31.5	3.2	
OB/GYN	92	0.2	0	0.0	−0.2	
Pediatric	12,273	24.4	73	19.6	−4.8	
Annual vaccine volume						
Highest (>10,000)	15,332	30.5	142	38.2	7.7	<0.0001
Extra high (2001−10,000)	17,624	35.1	130	35.0	−0.1	
High (1000−2000)	8559	17.1	66	17.7	0.6	
Medium (401−999)	5488	10.9	21	5.7	−5.2	
Low (≤400)	3207	6.4	13	3.5	−2.9	

## Data Availability

Data underlying this study are proprietary and are owned by VaxCare LLC and requests for access should be addressed to that organization. Because of their commercial and proprietary nature, the raw patient-level data are not publicly available. De-identified, aggregated summary data supporting the findings reported in this manuscript are available from the corresponding author.
